# Round the Hospitals

**Published:** 1917-01-27

**Authors:** 


### ROUND THE HOSPITALS.

THE event of the week has been the Special General Meeting of the members of the Royal British Nurses' Association. It was held in the large lecture-hall of the Royal Society of Medicine, 1 Wimpole Street, W., and was more largely attended than any previous meeting held there. It was interesting to note the 350 members or more, many of whom had come to the meeting at great personal sacrifice, representing practically every portion of the United Kingdom. The Lady is reported to have circulated the statement that the members in favour of amalgamation were the old nurses—*i.e.* presumably of no importance, as their working days were done. The members who attended were a splendid answer to this ill-natured libel, for they presented an appearance of energy, up-to-dateness, keen interest, and sober resolve which could not have been exceeded by any gathering of trained nurses in this or any other country. It is pitiable to have to deal with contemptible slanders of this description, but they have become the current coin of the party of "Divide," and in the interests of British nurses it is desirable that they should be nailed one by one to the counter and disposed of promptly. In this connection it is noteworthy that Mrs. Bedford Fenwick's speech at the meeting was limited to the words, "Hear, Hear" (see p. 354), which those who refer to Dr. Griffith's speech, where they occurred, will perceive, demonstrate an assumed or actual ignorance of plain facts which is remarkable or unworthy in either event. It is a thousand pities that one, who has shown herself capable of high ideals and better things, should persistently pursue a course which, as she consciously demonstrated, has reduced her to an impotence, that exhibits her lack of authority with the great body of British nurses.

---

THE keenness and spirit exhibited by the nurse members of the Royal British Nurses' Association were remarkable. One nurse on night duty in a distant hospital came off duty in the morning, got into the train which brought her to the meeting,

**ROUND THE HOSPITALS**— (*continued*).

and commenced her return journey immediately afterwards to enable her to be in time to go on duty again the same night. That meant for her a night out of bed and thirty-six continuous hours without rest save, such as she could get when travelling. One member came, as her happy little speech at the meeting demonstrated, from " near Land's End," whilst others travelled all the way from Scotland, Ireland, and various parts of England and Wales in order to vote for the establishment of the Royal British College of Nursing. It will be seen, therefore, that if, as one doctor maintained, nurses as a whole are woefully indifferent to their own interests, and can with difficulty be induced to record their vote on any given matter, the average member of the Royal British Nurses' Association is free from such faults, and deserves every credit for the energy and spirit she manifested on the 18th instant. We hope this spirit will now find expression in fuller measure by every one of the four or five thousand nurses who are members writing to the Editor of *The Nursing Mirror* for Application Forms for Registration, that each may act as a Recruiting Sergeant and secure that every trained nurse of her acquaintance shall fill in a form and send in her name to be entered on the College Register without delay. Each one, who wishes to become a Recruiting Sergeant for the College Register, can procure all the forms of application she may need by writing a line to the Editor of *The Nursing Mirror*, 28 Southampton Street, Strand, W.C. The sooner this action is taken the sooner will Registration by Statute be secured. The profession generally has been recruiting vigorously during the past six weeks. Now is the time for the Nurses of the Royal British Nurses' Association to set to work as Recruiting Sergeants and manifest their influence and authority in defence of their profession.

---

We regret that Dr. Griffith should not have made himself fully acquainted with the analogy that attaches to the plan of electing the members of the Central Midwives Board and those of the College Council under Mr. Stanley's Bill. The same principle underlies both cases. The Central Midwives Board under the Act consists of persons appointed by the Privy Council, by the Royal Colleges of Physicians and Surgeons, the Society of Apothecaries, the Midwives' Institute, by County Councils, Queen Victoria's Jubilee Institute, and the Royal British Nurses' Association. The Council of the Royal British College of Nursing, under Mr. Stanley's Bill, will consist of persons appointed by the Privy Council and any Government Department; and of representatives of the nurse-training schools, of the medical profession, and of the nurses on the Supplemental Registers; provided that not less than two-thirds of the members shall be elected by the nurses on the General Register under the Bill. It would have removed misapprehension if the chairman had put Dr. Griffith right, for the point raised was important, and it will be seen could have easily been met by a simple statement of the actual terms of Mr. Stanley's Bill. The final wording of the amended Charter has to be agreed with the Privy Council, and there should be no difficulty in making this fulfil every condition best calculated to promote the highest interests of the Nursing Profession, while it gives to individual nurses on the Register a Status, Protection, and Authority, the absence of which has given rise to some heartburning and much misapprehension in recent years.

---

The meeting on the 18th instant demonstrated the unity which now happily characterises the great body of British nurses in support of the Royal British College of Nursing and its Register, which should speedily be granted State Recognition and secure for the Nursing Profession in these islands a Position which should make it the envy and admiration of the world. We congratulate the authorities of the Royal British Nurses' Association and the Hon. Arthur Stanley, M.P., the chairman of the College of Nursing, who has now become the chairman of the Royal British College of Nursing, on the complete success and unanimity which the great meeting at the Royal Society of Medicine on the 18th instant proved to demonstration.

---

Another proof of the widespread interest excited by the Royal British College of Nursing throughout the profession of British nurses is the awakened interest exhibited by trained nurses in Ireland. In addition to the important meeting in Dublin on the 27th instant, of which we gave full particulars last week, there will be another large meeting in Belfast on Monday, the 29th instant, at 7 p.m., at the Royal Victoria Hospital, to discuss the College of Nursing and its Register. Professor J. A. Lindsay, M.A., M.D., has kindly consented to take the chair, and Miss Cox-Davies, and Miss Rundle the secretary of the College, will be amongst the speakers.

---

Already the effects of amalgamation and the birth of the Royal British College of Nursing are manifesting themselves throughout the country. An important meeting was held at Leeds on the 19th instant, when Alderman Lupton, ex-Lord Mayor and one of the most zealous and valuable of hospital workers and authorities in the provinces, made an interesting speech, which will be found on page 348. The Hon. Arthur Stanley was at his best. He enforced the important bearings of the amalgamation; he reminded the nursing profession that if the nurses objected to business management in their affairs they could drop the laymen by voting them off the Council in the middle of 1918. He showed the importance and value of laymen on the Council by insisting that the College could not do the work unless it had a satisfactory endowment. He then made the splendid announcement that he had sounded two great organisations which had collected £700,000 for the Red Cross, and found that each of them wanted to have the privilege of raising all the money required for the purposes of the College of Nursing. *Mr. Stanley* further demonstrated the value of the lay element by adding that he proposed to bring the representatives of the two organisations together, and to endeavour to get them to unite forces, "*to secure the College an endowment of which it might be proud.*" Miss Swift insisted that the College would be a democratic institution. We felt it was a great privilege to have her on the platform, for she has done such splendid organising work through the Red Cross (Joint Committee) throughout the war, and before it at Guy's and elsewhere, that no words can be too strong to record the nation's obligation to her for invaluable and lifelong service. Sir Berkeley Moynihan, the great Leeds surgeon, gave his blessing to the College. He declared that the British medical profession realised, whatever might be said of the international standard of its physicians and surgeons, that there was only one opinion in the whole world with regard to the international standard of British nurses.

